# Chronic pain-related consultations to the emergency department of children with complex pain conditions: A retrospective analysis of healthcare utilization and costs

**DOI:** 10.1080/24740527.2022.2070840

**Published:** 2022-06-17

**Authors:** Michelle Stoopler, Manon Choinière, Annabelle Nam, André Guigui, Laurel Walfish, Nada Mohamed, Marie Vigouroux, Victor-Hugo González-Cárdenas, Pablo Ingelmo

**Affiliations:** a Faculty of Medicine and Health Sciences, McGill University, Montreal, Quebec, Canada; b Department of Anesthesiology and Pain Medicine, Université de Montréal, Montreal, Quebec, Canada; c Research Center of the Centre Hospitalier de l’Université de Montréal, Montreal, Quebec, Canada; d CPSS - Performance Improvement Department McGill University Health Centre, Montreal, Quebec, Canada; e Department of Anesthesia, Montreal Children’s Hospital, Edwards Family Interdisciplinary Center for Complex Pain, Montreal, Quebec, Canada; f Faculty of Dentistry, McGill University, Montreal, Quebec, Canada; g School of Medicine, Fundación Universitaria de Ciencias de la Salud (FUCS), Bogotá, Colombia; h Department of Anesthesia, Los Cobos Medical Center; Department of Anesthesia, Pain & Palliative Care, Hospital Universitario de la Samaritana, Bogotá, Colombia; i Research Institute, McGill University Health Centre, Montreal, Quebec, Canada; j Alan Edwards Centre for Research on Pain, McGill University, Montreal, Quebec, Canada

**Keywords:** chronic pain, pediatric, emergency department, interdisciplinary treatment, costs

## Abstract

**Background:**

There is limited information regarding the effects of pediatric chronic pain management on the number and cost of chronic pain–related emergency department (ED) consultations.

**Aim:**

This retrospective study aimed to evaluate the number and costs of chronic pain–related ED consultations of children and adolescents with chronic pain conditions at the Montreal Children’s Hospital (MCH).

**Methods:**

Charts of patients followed by the Edwards Family Interdisciplinary Center for Complex Pain (CCP) of the MCH between April 2017 and December 2018 were reviewed. ED consultations, specialist consultations, medication prescriptions, hospital admissions, and outpatient consultation referrals were assessed for the period of 1 year before and after the patients’ first consultation with the CCP. Associated costs were also calculated.

**Results:**

One-hundred sixty-eight patients were included in the analysis. Fifty-one percent consulted the ED and had 151 chronic pain–related ED consultations within 1 year before their initial CCP consultation. In the year following their first CCP consultation, 52 patients (31%) consulted the ED, of which 24 consultations were chronic pain–related (84% reduction). There was an 81% reduction in the costs associated with chronic pain–related ED consultations within 1 year after CCP management. In addition, there was a significant reduction in ED interventions within 1 year after CCP management, though there was no change in medication prescriptions, hospital admissions, or subspecialist consultations.

**Conclusion:**

Children and adolescents with chronic pain conditions had fewer chronic pain–related ED consultations within 1 year after the first evaluation by an interdisciplinary center for complex pain, contributing to reduced ED costs.

## RESUMEN

**Contexte** : L'information sur les effets de la prise en charge de la douleur chronique pédiatrique sur le nombre et le coût des consultations liées à la douleur chronique au service des urgences est limitée.

**Objectif** : Cette étude rétrospective visait à évaluer le nombre et le coût des consultations liées à la douleur chronique des enfants et des adolescents souffrant de douleur chronique au service des urgences de l’Hôpital de Montréal pour enfants.

**Méthodes** : Les dossiers de patients suivis par le Centre interdisciplinaire de la famille Edwards pour la douleur complexe (CCP) de l’Hôpital de Montréal pour enfants entre avril 2017 et décembre 2018 ont été examinés. Les consultations au service des urgences, les consultations de spécialistes, les ordonnances de médicaments, les admissions à l'hôpital et les références pour consultation externe ont été évaluées pour la période d’un an avant et après la première consultation des patients auprés du CCP. Les coûts associés ont également été calculés.

**Résultats** : Cent soixante-huit patients ont été inclus dans l'analyse. Cinquante et un pour cent ont consulté le service des urgences dans le cadre de 151 consultations liées à la douleur chronique au service des urgences au cours de l'année précédant leur première consultation au CCP. Dans l'année suivant leur première consultation au CCP, 52 patients (31 %) ont consulté le service des urgences. Vingt-quatre de ces consultations étaient liées à la douleur chronique (une réduction de 84 %). Une réduction de 81 % des coûts associés aux consultations liées à la douleur chronique au service des urgences a été observée dans l'année suivant la prise en charge par le CCP. En outre, une réduction significative des interventions du services des urgences dans l’année suivant la prise en charge par le CCP a été observée, bien quéil néy ait pas eu de changement dans les ordonnances de médicaments, les admissions à léhôpital ou les consultations de sous-spécialistes.

**Conclusion** : Les enfants et les adolescents souffrant de douleur chronique ont consulté le service des urgences pour la douleur chronique moins souvent dans l'année suivant la première évaluation par un centre interdisciplinaire pour la douleur complexe, contribuant ainsi à réduire les coûts du service des urgences.

## Introduction

People who live with a chronic pain condition, including children and adolescents, often present to the emergency department (ED).^1^ This most likely occurs in the context of the chronic, relapsing course of chronic pain, which is often associated with pain crises and exacerbations. However, it has been shown that the pain management approach in the ED is not properly tailored to treating people with chronic and complex pain.^1^ In addition, frequent ED consultations due to poorly managed chronic pain contribute to ED overcrowding, prolonged wait times, and increased health care costs and do not adequately address the underlying pain disorder responsible for the pain crises. Tumin et al. assessed the prevalence of pediatric chronic pain and the use of health care services. They found that chronic pain diagnoses were associated with an increased use of ED services and were independently associated with increased medical expenditures.^2^

Specialized interdisciplinary chronic pain treatment facilitates promptly administered, effective, and patient-centered care. It also allows for an evaluation of the individual’s specific pain characteristics and thus the crafting of a pain management plan that is specifically tailored to the individual’s pain experience.^3–5^ Studies have been conducted internationally examining health care utilization and costs for pediatric patients with chronic pain.^2,5–13^ Many such studies have demonstrated a significant reduction in utilization of health care resources and costs following pain management provided by interdisciplinary chronic pain centers.^6–8,10,12,13^ Campbell et al. showed a significant reduction in physician remuneration claims across various health care service departments within the first year of treatment by an outpatient interdisciplinary chronic pain management program, with further decreases over the subsequent 5 years.^6^ A study by Mahrer et al. found a significant decrease in ED consultations and in hospital and insurance cost savings, even when taking into account costs of pain clinic services, within the year following chronic pain management program admission.^10^

Inpatient, intensive interdisciplinary pain treatment programs have also been associated with significant decreases in health care utilization and costs.^7,8,11,13^ Evans et al. noted an overall decrease in health care utilization but did not find a statistically significant reduction in ED usage,^7^ whereas Ruhe et al. found a decrease in health care utilization but no statistically significant difference in health care costs.^11^ These studies included self-report questionnaires, semistructured interviews, physician remuneration and health insurance claims, among other measures used that may have associated biases. In addition, the analysis time periods and types of pain management programs being examined were variable.

Though there are some studies examining healthcare utilization and costs for pediatric patients with chronic pain,^2,5–13^ only four studies specifically evaluated the costs associated with ED consultations.^7,9,10,13^ However, there is a scarcity of research quantifying the impact of the treatment of interdisciplinary complex pain centers specifically on the number of consults and services provided by the ED for children and adolescents with complex pain conditions. Moreover, only a few of these studies provided a detailed analysis of the types of consultations seen in the ED (chronic pain related versus non-chronic pain related) or the costs associated with the different types of chronic pain conditions or differentiated between the types of costs (direct versus indirect) associated with ED consultations. This particular analysis is relevant given that up to 11% of health care costs associated with treating pediatric patients with chronic pain were related to the high number of ED consultations.^14^

The aim of this study was to evaluate the number of ED consultations for patients followed by the Edwards Family Interdisciplinary Center for Complex Pain (CCP) of the Montreal Children’s Hospital (Quebec, Canada) within 1 year before and after the first evaluation by the CCP. We also evaluated the services provided by the ED, as well the fiscal analysis of associated hospital costs. We hypothesized that within 1 year after the first evaluation by the CCP, there would be a reduction in the number of consults to the ED as well as a decrease in ED-associated health care costs.

## Materials and Methods

### Population and Procedure

This study was approved by the McGill University Health Centre (MUHC) Research Ethics Board (2019-4670). Informed patient and/or parental consent was granted in the context of the nature of the study. It consisted of a retrospective chart analysis for children between the ages of 2 and 18 years who lived with a chronic pain condition and had been admitted to the CCP of the Montreal Children’s Hospital between April 2017 and December 2018. The chart analysis covered the year before and the year after the patients’ initial consultations with the CCP. The chart analysis was completed using two electronic medical record databases: the OACIS electronic medical record used in the MUHC institutions and the CCP’s internal patient database. These databases systematically document all visits to the ED at the MCH, thus capturing all consultations a patient had at the institution.

### Description of the CCP

The outpatient program of the Edwards Family Interdisciplinary Center for Complex Pain focuses on optimizing physical and psychological function, normalizing sleep and social function, and increasing levels of activity, while assisting with the management of the pain. The core team at each evaluation includes a nurse, psychologist, social worker, physiotherapist, clinical fellow, and anesthesiologist specialized in pain management. During an initial interdisciplinary face-to-face interview, a formal evaluation of pain intensity and the impact of the pain condition on the patient’s physical function, mood, sleep, academic performance, and social life, as well as the patient’s expectations and goals, is conducted. A detailed physical exam is conducted by a pain specialist and a physiotherapist. A psychologist, a social worker, and a nurse clinician interview the patient/caregiver independently. At the end of the evaluation, the patient and their parents/caregivers discuss the diagnosis and a personalized treatment plan with the interdisciplinary team. This treatment plan may include medications, physiotherapy, psychology services, nursing services, social services, or interventional procedures, among various other modalities that are cohesively used to provide a multidimensional approach to reduce pain disability. The interdisciplinary program also includes cognitive behavioral therapy and individual and group outpatient physiotherapy sessions. Medical treatments performed are based on the results of quantitative sensory testing and conditioned pain modulation evaluations, in addition to physical examination findings.^15^ Patients are typically seen for multidisciplinary follow-up visits every 3 months, though they can be seen sooner if issues are identified or changes to the treatment plan are needed. Patients and parents can consult the CCP nurse clinicians during working hours using a dedicated phone number provided to them. The social workers, physiotherapist, and psychologists can individually reach the nurse clinicians and physicians through a team chat, allowing for faster and more efficient communication within the team. The team discusses the evolution of specific cases during three multidisciplinary team meetings per week. Finally, the CCP team provides a 24/7 on-call physician consultation service accessible to the ED and other specialties within the MCH.

Overall, more than 60% of patients assisted by the CCP spent less than 1 year in the ambulatory program (mean treatment time 257 days, 95% confidence interval [CI] 223 to 291). The costs of evaluations, treatment, and follow-up care provided by the CCP, as well as all consults and treatments provided by the ED, are entirely covered by the Quebec public health system.

### Measures

The primary endpoints of this study were the number of chronic pain–related ED consultations and their associated costs for children and adolescents followed by the CCP. The number of ED consultations within the study period was tabulated by performing a retrospective chart analysis and identifying all ED consultations within the analysis period for each individual. These consultations were then further classified as being “chronic pain related” or “non-chronic pain related.” Chronic pain–related consults were defined according to the *International Classification of Diseases* definition of chronic pain, which is pain present or recurring for greater than 3 months.^16^ In practice, a chronic pain–related consult should be linked to a specific chronic pain condition that may or may not be associated with the diagnosis established by the CCP. Examples of chronic pain–related consultations include a patient with migraines presenting for severe headache or a patient with chronic widespread pain presenting for a musculoskeletal pain crisis. Examples of non-chronic pain–related consultations are those for acute trauma, acute infection, new-onset seizures, and surgical consults, among others. Three team members (M.S., N.M., L.W.) independently categorized consults as “chronic pain related” or “non-chronic pain related.” Disagreements were resolved by cross-checking of both databases by the first and last authors (M.S., P.I.).

To evaluate the costs associated with these ED visits, the Coût Par Parcours de Séjours et Soins (CPSS) team of the MUHC used the Power Performance Manager system,^17^ an international tool used to calculate health care costs. This tool extracts raw data such as patient admissions, transfers, medical imaging, pharmacy, laboratories, and other costs from the MUHC’s internal clinical database. The CPSS team then performs manual calculation of these costs and specific services. Power Performance Manager then allocates these care costs directly to patients using different weights such as by visit, duration of care, and costs of specific supplies, among others. The indirect operating costs of the hospital, such as administration, housekeeping, laundry, building services, and security are also allocated to the “patient care” departments using their services based on internal allocation statistics (e.g., worked hours, expenses) that assign the cost to patients as “indirect” costs. The costs tabulated by the CPSS team presented in this study represent only the costs incurred and covered by the Montreal Children’s Hospital budget. The data analyzed in this study did not include the physicians’ salaries, which are covered by Régie de l’assurance maladie du Québec.

The secondary endpoints of this study include the services provided to pediatric patients with chronic pain within the ED, laboratory and imaging tests ordered, specialist consultations provided, medications received, hospital admissions, outpatient consultation referrals, and their associated costs.

### Statistical Analysis

Results are expressed as frequencies (*n*) and proportions (%). Median (and 25%–75% interquartile range, IQR) or mean (SD) were used according to the normality distribution as assessed by the Shapiro-Wilk or Kolmogorov-Smirnov test.

Comparisons between the year before and after the patients’ initial consultations with the CCP were made using Fisher’s exact test for dichotomous variables (patients consulting the ED, patients consulting due to chronic pain, diagnosis of chronic pain). Also, a two-sided Student’s *t* test or Mann-Whitney *U* test was used for continuous variables (number of ED consultations, number of chronic pain–related consultations, number of interventions, medication prescriptions, specialist consultations, hospital admissions, direct costs and indirect costs) according to the test of normality. Number of ED consultations and reconsultations was described and analyzed before and after the first CCP consultation. Average number of ED consultations and reconsultations were compared by Mann-Whitney *U* test. Likewise, the percentage of ED reconsultations before and after first CCP consultations were compared (using chi-square test and Fisher’s exact test). Both ED consultation number and costs were analyzed under diagnosis stratification by group (chronic primary and secondary pain) and specific diagnosis (chronic musculoskeletal pain, chronic widespread pain, chronic postsurgical/posttraumatic pain, chronic headache and orofacial pain, chronic neuropathic pain, chronic visceral pain). Relative risk (RR) and 95% CIs were also calculated. A two-tailed *P* value <0.05 was considered statistically significant. All analyses were performed with IBM SPSS statistics software v25.^18^

## Results

We analyzed the data of 168 individuals (80% female) with a median age at time of first consultation with the CCP of 15 years old (25%–75% IQR = 13–16). The race of the included patients was predominantly white (*n* = 151, 90%). Due to the low frequency of some racial groups, races typically identified by Statistics Canada as a visible minority group (Indigenous, South Asian, black, Latin American, Arab, and mixed race) were collapsed into a single category that included 17 patients (10%). The diagnoses of the individuals included in the analysis are summarized in Table 1, with 96 patients (57%) having a chronic primary pain diagnosis and 72 patients (43%) having a chronic secondary pain diagnosis.

**Table 1.** Chronic pain diagnoses of patients included in the study

Pain diagnosis	*n*	(%)
Chronic primary widespread pain	44	26
Chronic secondary musculoskeletal pain	36	21
Chronic primary musculoskeletal pain	31	18
Chronic postsurgical/posttraumatic pain (chronic secondary pain)	17	10
Chronic secondary neuropathic pain	14	8
Chronic primary headache and orofacial pain	12	7
Chronic secondary headache and orofacial pain	3	2
Chronic primary visceral pain	9	5
Chronic secondary visceral pain	2	1

Data are presented as number of patients (*n*) and percentages (%).

### ED Consultations

There was a significant reduction in both the total number of ED consultations (46%) and specifically in the number of chronic pain–related ED consultations (84%) within the year after the first consultation with the CCP. The mean number of consults per patient was 2.8 ± 0.5 within 1 year before the first evaluation by the CCP and 2.5 ± 0.4 within 1 year afterward. In addition, there was a meaningful reduction in the number of chronic pain–related ED consults from 151 consults within 1 year before CCP evaluation to 24 consults within 1 year after (*P* < 0.01), as outlined in Table 2. The median number of reconsultations for chronic pain–related ER visits was 3 (IQR = 2 to 6) within 1 year before and 0 (IQR = 0 to 1) within 1 year after the first CCP consultation (*P* < 0.0001).

**Table 2.** Number of consultations to the ED and number of patients consulting the ED within 1 year before and 1 year after initial evaluation by the CCP

	Within 1 year before CCP	Within 1 year after CCP	Difference, *n* (%)	RR (95% CI)
ED visits	242	131	111 (46)**	0.66 (−1.11 to −0.21)
Chronic pain–related ED visits	151	24	127 (84)*	0.76 (−1.03 to −0.48)
Patients consulting the ED, *n* (%)	86 (51)	52 (31)	34 (40)*	0.61 (0.43–0.81)
Patients consulting due to pain, *n* (%)	65 (39)	17 (10)	48 (74)*	0.26 (0.15–0.43)
Patients consulting due to primary chronic pain, *n* (%)	37 (39)	10 (10)	27 (73)*	0.34 (0.17–0.65)
Patients consulting due to secondary chronic pain, *n* (%)	28 (39)	7(10)	21 (75)*	0.25 (0.11–0.54)

Data are presented as number of consults, number of patients, %, RR and 95% CIs.

*P < 0001. **P = <0.01.

### Patients Consulting the ED

There was also a meaningful reduction in the total number of patients followed by the CCP who consulted the ED within 1 year after initial CCP consultation and in the number of patients consulting specifically due to chronic pain, the latter of which decreased from 65 patients to 17 patients. The reduction in the proportion of patients consulting the ED and in the number of ED consultations was similar among patients with primary and secondary pain conditions (Table 2).

There was meaningful reduction in patients with chronic widespread pain, chronic musculoskeletal pain (both primary and secondary), and chronic primary headaches and orofacial pain consulting the ED due to chronic pain–related problems (Table 3).

**Table 3.** Patients consulting the emergency department due to chronic pain–related concerns within 1 year before and after initial evaluation by the CCP

Pain diagnosis	Patients consulting within 1 year before CCP, *n* (%)	Patients consulting within 1 year after CCP, *n* (%)
**Chronic primary pain**		
Chronic widespread pain	15/44 (34)	4/44 (9)*
Chronic musculoskeletal pain	10/31 (32)	2/31 (7)*
Chronic headache and orofacial pain	10/12 (83)	4/12 (33)*
Chronic visceral pain	2/9 (22)	0/9 (0)
**Chronic secondary pain**		
Chronic musculoskeletal pain	13/36 (36)	2/36 (6)*
Chronic postsurgical/posttraumatic pain	5/17 (29)	3/17 (18)
Chronic neuropathic pain	5/14 (36)	1/14 (7)
Chronic headache and orofacial pain	3/3 (100)	0/3 (0)
Chronic visceral pain	2/2 (100)	1/2 (50)

Data are presented as number of patients and %.

*P < 0.05.

### Course in the ED

During the chronic pain–related ED consultations. there was a meaningful reduction in the number of interventions for visits within 1 year following CCP management. There were no significant reductions in the number of medication prescriptions, subspecialist evaluations in the ED, hospital admissions, and referrals to ambulatory subspecialty consultations between the analyzed periods (Table 4).

**Table 4.** Interventions, medication prescriptions, hospital admissions, and subspecialist evaluations during and after the consultations to the ED

	Within 1 year before CCP	Within 1 year after CCP	Difference (%)
Interventions	127	51	60*
Prescription of medications during the ED admission	139	70	50
Admission to the hospital	19	15	21
Evaluation by other specialties in the ED	35	23	35
Consultation for an outpatient evaluation by another specialty after discharge from the ED	84	46	45

Interventions include imaging studies, nerve blocks, or other miscellaneous procedures. Data are presented as absolute numbers and net reduction (%) of interventions, medication prescriptions, hospital admissions, and subspecialist evaluations 1 year before and 1 year after initial consultation by the CCP.

*P < 0.0001.

### Costs

There was a significant reduction in the total costs associated with ED consultations within 1 year following the first evaluation by the CCP. The total cost of the ED consultations in the year before CCP consultation was CA$51,218, compared with CA$41,280 within 1 year after the first evaluation by the CCP (net reduction 19%, *P* < 0.0001). There was a significant reduction in the direct costs before (CA$35,992) compared with after (CA$28,801) the first evaluation with the CCP (net reduction 20%, *P* < 0.0001). The difference between the total indirect costs before (CA$15,226) and after (CA$12,479) the first evaluation with the CCP was not significant (*P* > 0.05).

There was an 81% net reduction in the costs associated with chronic pain–related ED consultations when comparing the total cost of these consultations within 1 year before (CA$34689) and within 1 year after (CA$6634) the first evaluation by the CCP (*P* < 0.001) The mean chronic pain–related ED consultation cost was CA$228 ± CA$167 within 1 year before and CA$276 ± CA$164 within 1 year after the first evaluation by the CCP.

## Discussion

This study evaluated the number of ED consultations, stratified into chronic pain–related and non-chronic pain–related consultations, services, and associated costs for pediatric patients with chronic pain within 1 year before and after the first evaluation by an outpatient interdisciplinary chronic pain management program. Within 1 year after the first evaluation by the CCP, patients with chronic pain conditions consulted the ED less often, independent of the cause for the consultation (i.e., chronic pain–related or non-chronic pain–related consultations). We found a meaningful reduction in the number of patients consulting the ED for those with both primary and secondary pain conditions. More specifically, patients with chronic widespread pain, chronic musculoskeletal pain, and chronic primary headaches and orofacial pain were found to have significant reductions in the number of consultations to the ED. There was a significant reduction in the number of interventions in the ED within 1 year following first CCP evaluation but not in the number of medication prescriptions, hospital admissions, and referrals to other specialties. Finally, the fiscal analysis showed a reduction in the total and direct costs associated with ED consultations as well as the costs associated with chronic pain–related ED consultations.

The decrease in chronic pain–related ED visits may be due to several reasons. These include the interdisciplinary nature of the CCP services, the patient-tailored treatment plan, the pain-specific management expertise of the CCP team, and the ongoing support for patients and parents provided by the CCP team. The CCP provides specific counseling to patients and their families regarding what to do in the event of pain crises that may have previously led them to visiting the ED. Patients managed by the CCP receive a clear action plan in the event of pain crises and anxiety or depressive mood and are instructed to consult the ED only if such measures fail to manage the pain.

The decrease in the total number of ED visits (i.e., including non-chronic pain–related ED consultations) observed within 1 year following the first CCP evaluation may also be due to improved communication with and access to health care professionals. The CCP team guides patients and their families on common health issues and appropriate treatment venues, including consults to family physicians and pediatricians. The high interprofessional connectivity within the CCP team may also prevent unnecessary consults to the ED. The three interdisciplinary team meetings per week may also help anticipate the need for urgent consults before they result in ED visits. The effective communication with the team members may improve the overall perception of inclusion in a safety network and the confidence of patients and families to manage their conditions without consulting the ED.

The significant reduction in ED interventions for chronic pain–related visits within 1 year following CCP management may also be due to several factors. The treating ED team can easily reach the CCP team nurses during working hours and the on-call CCP physician at all times for consultation and for advice regarding the personalized treatment of specific pain conditions. The access to a formal diagnosis through the patients’ electronic medical records may limit the need for extensive diagnostic workup and imaging to look for a source of pain. Finally, most patients have had previous imaging and laboratory studies performed during prior ED visits, which may preclude the need for additional tests.

We did not find significant reductions in the number of medication prescriptions, subspecialist evaluations in the ED, hospital admissions, and referrals to ambulatory subspecialty consultations between the analyzed periods. This may demonstrate that treating ED physicians were not changing their clinical management of pain crises despite outpatient management by the CCP. The impact of interdisciplinary chronic pain management on the medical decision making applied by ED physicians deserves further attention and research.

The significant reduction in direct costs but not indirect costs for chronic pain–related ED visits is also an expected finding. The indirect costs represent operating costs incurred by the hospital, which are likely to remain constant because they are allocated to the “patient care” departments using their services based on internal allocation statistics and then assigned to patient care costs. The most effective way to prevent indirect costs of ED consultations remains the prevention of the ED consults.

The similar reduction in the number of patients consulting the ED for those with primary and secondary pain conditions was also expected. Even if most patients’ treatment plans were tailored to their individual pain experience, most patients receive multimodal interventions within the interdisciplinary pain treatment program. The mean treatment time with the CCP is 9 months, with more than half of patients being discharged from the program within 1 year. The timing of the benefits of CCP interventions is likely to similarly impact patients with primary and secondary pain conditions.

The reduction in ED consultations represented a decrease in utilization of certain health care resources and provided a reduction of the burden of ED overcrowding, a serious health care issue in pediatric emergency rooms. It also saved the health care system economic resources. However, the cost savings related to ED consultations were not compared with data regarding the costs associated with CCP management, thus precluding us from commenting on the cost savings in comparison to CCP costs for managing these patients. Hospital administrators may interpret these data as demonstrating that outpatient interdisciplinary chronic pain management programs may represent a promising avenue to reducing the burden of pediatric chronic pain and decreasing chronic pain–related ED overcrowding and associated costs. This may provoke changes to the funding of such programs, though future studies evaluating its cost efficiency are still necessary. In addition, this may promote more training of ED physicians and pediatric pain specialists, because this is a relatively small subspecialty not available in all communities.

Our findings are consistent with previous studies that have examined the impact of interdisciplinary pediatric chronic pain management programs on health care utilization and associated costs.^2,5–13^ Our data complement the results of Campbell et al.^6^ and Mahrer at al.,^10^ who also examined outpatient-based programs. We included hospital- and medication-related costs and stratified ED consultations into chronic pain related versus non-chronic pain related, providing further insights on the effect of specialized chronic pain management on chronic pain–specific ED consultations. We consider this stratification in ED consultations a strength of our study.

Our study findings are also consistent with studies of intensive, inpatient-based interdisciplinary pain management programs, such as those evaluated in the studies by Evans et al.,^7^ Hechler et al.,^8^ Ruhe et al.,^11^ and Lopez Lumbi et al.^13^ These studies showed a decrease in overall health care utilization, which they examined through measures such as frequency of inpatient hospitalizations, pain-related ED visits, and specialist consultations, among other endpoint measurements examined over varying time periods. These studies also showed a reduction in health care costs as examined through measures such as subjective financial burden, health insurance claims, and cost estimate calculations using health care cost data tools. Similar to these studies, our study showed a decrease in health care utilization, specifically ED utilization, and costs following management by a specialized pain management program. However, it is difficult to compare our findings to the findings of these studies because the nature of intensive inpatient-based pain management programs is different from that of the outpatient-based CCP.

Though many of the previous studies demonstrated a decrease in health care utilization and costs following interdisciplinary chronic pain management services, a study by Wager et al. demonstrated that though pediatric patients with chronic pain treated with intensive inpatient chronic pain management had a high likelihood of recovering from their chronic pain condition by adulthood, they continued to display increased health care utilization irrespective of whether they were still experiencing chronic pain or not.^19^ This highlights that pediatric patients with chronic pain are likely to experience increased healthcare utilization even several years following management of their pain, thus contributing to high healthcare costs.

### Strengths and Limitations

This study has several strengths. Firstly, it is based on a relatively large sample size of patients analyzed in daily clinical practice. Secondly, it provides the stratification of ED consultations into chronic pain related versus non-chronic pain related for each ED consultation. With this information, we can further tease out the changes specifically to chronic pain–related ED consultations and costs that we would expect to be affected by CCP management. The use of a real-time, high-fidelity systematic database ensures a high degree of reliability of the results and avoidance of biases such as recall bias that can be observed with other types of methodologies evaluating health care utilization. In addition, the analysis period of 1 year before and after pain management program intervention is consistent with the average treatment period of patients in the CCP program. Our data provide concrete fiscal evidence that governmental organizations and donors can refer to when making decisions regarding funding of health care services, such as chronic pain management programs.

This study had several limitations. First, the analysis period can be viewed as relatively short compared to some other studies that utilized a longer analysis period of several years. Second, we cannot be certain that the reduction in ED consultations is the result of interventions by the CCP, because we did not include a comparator group of patients with chronic pain who were not managed by the CCP. As such, it was not possible to estimate the potential effects of natural healing or improvement of patients with chronic pain conditions. A control group of patients on a waiting list may help to evaluate the hypothesis of the potential natural improvements associated with a reduction in the number of ED consults. Almost all studies evaluating the cost of health care utilization in children with chronic pain compared the costs before and after an intervention^6–13^ or described the cost utilization of patients with chronic pain conditions without including control groups.^2,14^ Hechler et al. included a waiting list group as a control. However, due to the waitlist design, the original randomization did not have any effect on the follow-up analyses of the economic impact of the treatment that was not calculated.^8^ Another limitation to consider is that the database used captured only visits to the one institution (MCH) and did not capture any visits to other clinics or hospitals a patient may have consulted during the same period. Moreover, the costs analyzed in this study represent costs incurred by the Montreal Children’s Hospital in relation to the care provided. We did not include physician salaries, which are covered by another governmental office (Régie de l’assurance maladie du Québec) and are not available for analysis. This limitation makes our cost data results difficult to compare with those of other institutions that include physician expenses in their analyses. In addition, the health care costs tabulated in this study did not include other health care–related costs that may have been incurred by patients and their families, including but not limited to costs associated with travel to and from the hospital, private health care, or alternative medicine costs, among other possible costs. Finally, the limited diversity in the sample population, given that the majority of included patients are white, is a limitation to generalizability of the results to patients of varying backgrounds.

Future avenues in this field of research include evaluating the efficacy of interdisciplinary pediatric complex pain management using a control group of chronic conditions not associated with chronic pain. In addition, cost analysis over a more extended period of time would provide a longitudinal analysis on the transition to adult care. Further evaluation of which chronic pain diagnoses are correlated with the greatest utilization of healthcare services would be useful as well to understand where to centralize pain management improvement efforts. Further research is also necessary to evaluate for confounding factors contributing to the reduction in ED consultations following management by a complex pain center.

In conclusion, pediatric patients assisted by a complex pain center visited the ED less frequently within 1 year following the first evaluation by an interdisciplinary complex pain program. The reduction in the number of consultations was associated with meaningful reductions in the use of healthcare resources and costs.
